# Population Fluctuations and Synchrony of Grassland Butterflies in Relation to Species Traits

**DOI:** 10.1371/journal.pone.0078233

**Published:** 2013-10-22

**Authors:** Markus Franzén, Sven G. Nilsson, Victor Johansson, Thomas Ranius

**Affiliations:** 1 Department of Community Ecology, UFZ, Helmholtz Centre for Environmental Research, Halle, Germany; 2 Department of Biology, Biodiversity and Conservation Biology, Lund University, Lund, Sweden; 3 Department of Ecology, Swedish University of Agricultural Sciences, Uppsala, Sweden; 4 Department of Ecology, Environment and Plant Sciences, Stockholm University, Stockholm, Sweden; New England Aquarium, United States of America

## Abstract

Population fluctuations and synchrony influence population persistence; species with larger fluctuations and more synchronised population fluctuations face higher extinction risks. Here, we analyse the effect of diet specialisation, mobility, length of the flight period, and distance to the northern edge of the species’ distribution in relation to between-year population fluctuations and synchrony of butterfly species. All butterfly species associated with grasslands were surveyed over five successive years at 19 grassland sites in a forest-dominated landscape (50 km^2^) in southern Sweden. At both the local and regional level, we found larger population fluctuations in species with longer flight periods. Population fluctuations were more synchronous among localities in diet specialists. Species with a long flight period might move more to track nectar resources compared to species with shorter flight period, and if nectar sources vary widely between years and localities it may explain that population fluctuations increase with increasing flight length. Diet generalists can use different resources (in this case host plants) at different localities and this can explain the lower synchrony in population fluctuations among generalist species. Higher degree of synchrony is one possible explanation for the higher extinction risks that have been observed for more specialised species. Therefore, diet specialists are more often threatened and require more conservation efforts than generalists.

## Introduction

Theoretical and empirical studies suggest that larger fluctuations in population size increase the extinction risk [1]. The amplitude of population fluctuations varies greatly among species [2,3], and is affected by the degree of synchrony among different parts of the population; the lower the degree of synchrony among local subpopulations, the more restricted the fluctuations in the overall population [4]. Populations that are spatially synchronised over large distances are generally more extinction-prone, because then several subpopulations may go extinct at the same time without being rescued by immigration from other subpopulations [5]. Spatial synchrony of population dynamics can be attributed to spatially correlated variation in weather [6], habitat quality [7], and mortality factors such as predation, parasitism, and diseases [8,9]. 

If particular species traits are related to the amplitude of population fluctuations and degree of synchrony, they may explain differences in population persistence between species. Species more specialised to specific habitats may fluctuate more because they are concentrated on localised resources which only occur in certain circumstances, for instance, microclimatic conditions. If individuals in every locality occur under similar conditions, the synchrony among subpopulations may increase, which would increase fluctuations. Species with a long flight period and more mobile species can be assumed to fluctuate more, at a local scale as they have more time to move among local patches, tracking the fluctuation of nectar resources, and at a regional scale as they may immigrate from surrounding regions to a higher extent [10]. Also populations of species close to their distribution limits are more influenced by extreme weather and have been shown to fluctuate more [11,12]. Population fluctuations have rarely been related to species traits [13,14]. This may be because fluctuations are difficult to measure in a way that allows comparison among species. Here we provide the first multiple-species examination of the associations between species traits and population fluctuations.

We analyse abundance data between five successive years for 31 resident butterfly species with different traits from 19 semi-natural grassland sites in a forest dominated landscape. We made between-species comparisons of population fluctuations and the synchrony of the population fluctuations. We tested the following hypotheses:

1 Population fluctuations and the degree of synchrony are higher among diet specialists compared with diet generalists.
2 Species with a long flight period and more mobile species fluctuate more than those with a shorter flight period and less mobile species.
3 Populations fluctuate more when species are close to the northern edge of their distribution range.

## Material and Methods

### Study landscape

**Figure 1:**
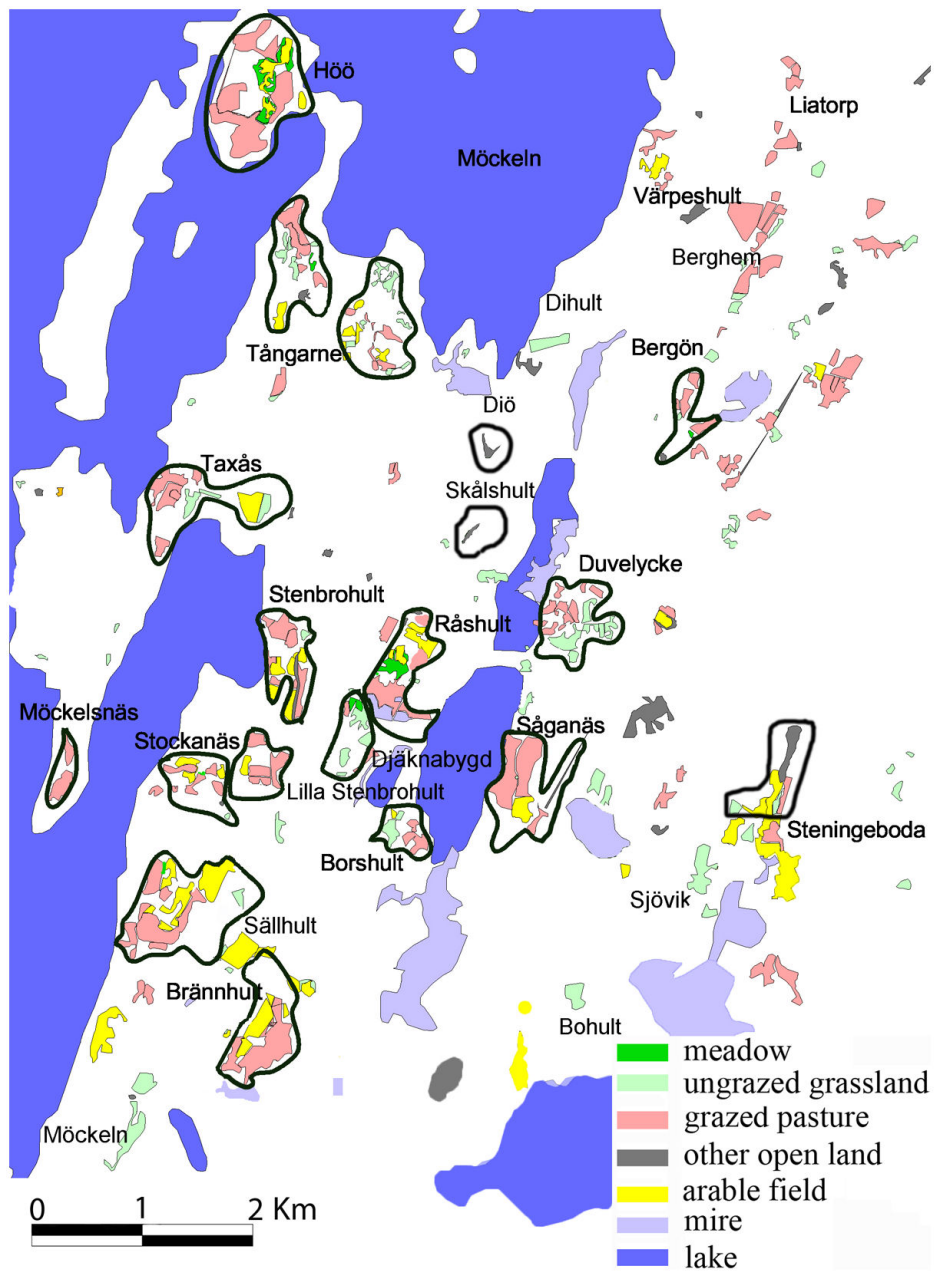
The study area in southern Sweden. The 19 surveyed sites are surrounded by thick lines and the white areas are forest.

The study was conducted in the parish of Stenbrohult, southern Sweden (56°37´N, 14°11´E). Within the study area, semi-natural grassland habitats comprising hay-meadows, grazed pastures, and recently abandoned pastures cover 5% of the land area. The surveyed sites were patches of semi-natural grassland, which are isolated by dense forest, mainly dominated by Norway spruce *Picea abies*. On most surveyed localities, grazing with cattle and horses was the dominant land use. Two of the surveyed localities were small abandoned grasslands resembling ruderal habitats. The study area is representative for the wider forest-dominated landscape of the region, consisting mainly of forests, mires and lakes. The study area cover approximately 50 km^2^ (9 km × 6 km; Figure 1) and is adjacent to the large lake Möckeln in the west and built-up areas and an exploited bog to the south. Coniferous forests dominate to the north and east. Thus, the landscape surrounding the study area has a low proportion of grasslands.

In each of the 19 surveyed sites, the whole area of semi-natural grassland was surveyed. The surveyed sites were separated by at least 100 m of woodland habitat (Figure 1). Herb-rich fields, field margins, road verges with a rich flora, and old hay meadows were classified as semi-natural grasslands. Arable fields recently (i.e., in the last 10 years) converted to fertilized grasslands were not sampled, but occupied a low proportion of our study area (1%). 

### Butterfly survey

Butterflies were surveyed from 2001 to 2005, using simplified transect walks modified from Pollard [15]. Each transect was walked slowly (about 1 km h^-1^) between 9 am and 5 pm in June and July, and between 10 am and 4 pm in April, May, August, and September. The transects criss-crossed all semi-natural grassland habitats at each surveyed site at intervals of approximately 20–30 m. The same survey density was applied regardless of the area of semi-natural grassland within each grassland site, and thus at larger sites longer transects were walked. Each survey walk required 1–7 hours depending on the size of the surveyed site. As far as possible, surveys were conducted on sunny days. Surveys were not performed in unfavorable weather conditions such as when it was raining (or within an hour of rainfall), when temperatures were lower than 17°C, or when there were moderate to strong winds (> 4 m s^-1^). 

All surveyed sites except one (surveyed 16 times each year) were surveyed from five to ten times a year (mean = 6), at regular intervals between the end of April and the beginning of September. This period encompasses the time frame when all butterfly species are active in the study area [16]. The number of days required to visit all sites was on average 17 days (min = 11, max = 31). Normally the butterflies in the study area have their peak of activity during at least 14 days (SGN unpublished data). All butterflies along the transects within a distance of 5 m of the observer were identified to species. Doubtful identifications were confirmed by closer inspection gained either by collecting the individual with a butterfly net and then releasing it, or by identifying the specimen through binoculars (10 × 40). Butterfly systematics followed Karsholt and Razowski [17]. No specific permissions were required for the study and the field studies did not involve endangered or protected species. The field work was carried out in accordance with the Swedish public rights of way, i.e. people have access to private land where no damage is likely to result and prohibition would be unreasonable.

### Studied species and traits

We only included species for which it was likely that the majority of the individuals in the landscape reproduced in the surveyed grassland sites. This was based on information on their habitat for reproduction [18–20]. Consequently, butterfly species associated with marshland, bogs, and arable fields, and the two immigrants *Cynthia cardui* and *Vanessa atalanta* were excluded. 

To avoid bias from zeros and low means [3], for each species we only included those sites where the species was present during at least three of the sampling years [12] and for every site only those years when the species was present. Species with data from two or fewer sites were excluded from the analysis. After this filtering, 31 species remained, with on average 15.2 ± 4.1 (SD; range 3–19) sites analysed per species, and 4.29 ± 0.85 (SD; range 3–5) years analysed per species and site (Table S1). 

We included four traits as explanatory variables: mobility, length of flight period, diet specialisation, and distance to northern edge. Mobility values were according to Komonen et al. [21], who compiled mobility values based on expert opinions and literature. Length of the flight period was estimated from Svensson [22] as an average (in weeks) for southern Sweden. For species with two generations, we summed the flight periods. For species with overwintering adults (n = 6), we summed the lengths of the flight periods in autumn and spring, but excluded the winter (15 October to 15 March). Diet specialisation was classified as (i) diet specialists (monophagous and oligophagous species lumped together), with strong larval diet preference for one to three plant genera or (ii) diet generalists, with no strong preference for a certain plant species as larval diet. The classification was based on information in Kuussaari et al. [20], and observations of host plant use in the study area. The distance to the northern edge of the species’ range was measured in kilometres from the central point of the study area. The northern edge was the northernmost point of the distribution in Fennoscandia according to Eliasson et al. [19]. 

### Data analyses

Population fluctuations were assessed as coefficients of variation (CV henceforth) in abundance over years. CV is the ratio of the standard deviation to the mean of the abundance per year, and was calculated at both the local and the regional level (the mean CV for abundance data from every site calculated separately, and for pooled total abundances, respectively). Abundance per year was measured as the number of individuals observed divided by the number of survey events during the flight period of the species. For species with two separate flight periods per year, we used information only from the period with the highest abundance. We first analysed CV at the locality level based on species traits, using a generalised linear mixed model (GLMM) with site as a random factor (to control for possible site effects). We also included species abundance to control for the possibility that CV is larger in smaller populations [3,23]. Second, we analysed CV at the regional level (i.e. CV from all 19 sites pooled), based on species traits and abundance using a generalised linear model (GLM). Synchrony was assessed using Pearson correlation coefficients between each pair of localities for species’ annual abundances [6]. For each species, synchrony was calculated as a mean of the correlation coefficients from all locality pairs (it may vary between -1 and 1). We analysed synchrony based on species traits and abundance using a GLM. To explore the robustness of our result we also performed the analyses excluding species with two generations. This was performed because it is not straightforward how to estimate population fluctuations and the length of the flight period for species with two generations. 

The models were built based on AIC (Akaike’s Information Criterion) [24], by comparing all possible models with different combinations of the explanatory variables. The final model, for each of the three response variables, was the one with the lowest AIC. The statistics software R 2.12.1[25] was used for the analyses. Summary data for each species is presented in Table S1. 

## Results

Between-year population fluctuations (CV) were wider at the local scale compared with the regional scale (mean: 1.39 vs. 1.02). The species with the greatest fluctuations on the regional scale was the diet specialist *Polyommatus icarus* with a CV of 1.66, with abundances ranging from 5 individuals in 2001 to 77 in 2004 (Table S1). The diet generalist *Aphantopus hyperanthus* was the most stable species with a CV of 0.56, with abundances ranging from 5372 in 2001 to 15 900 individuals in 2004 (Table S1). 

**Figure 2:**
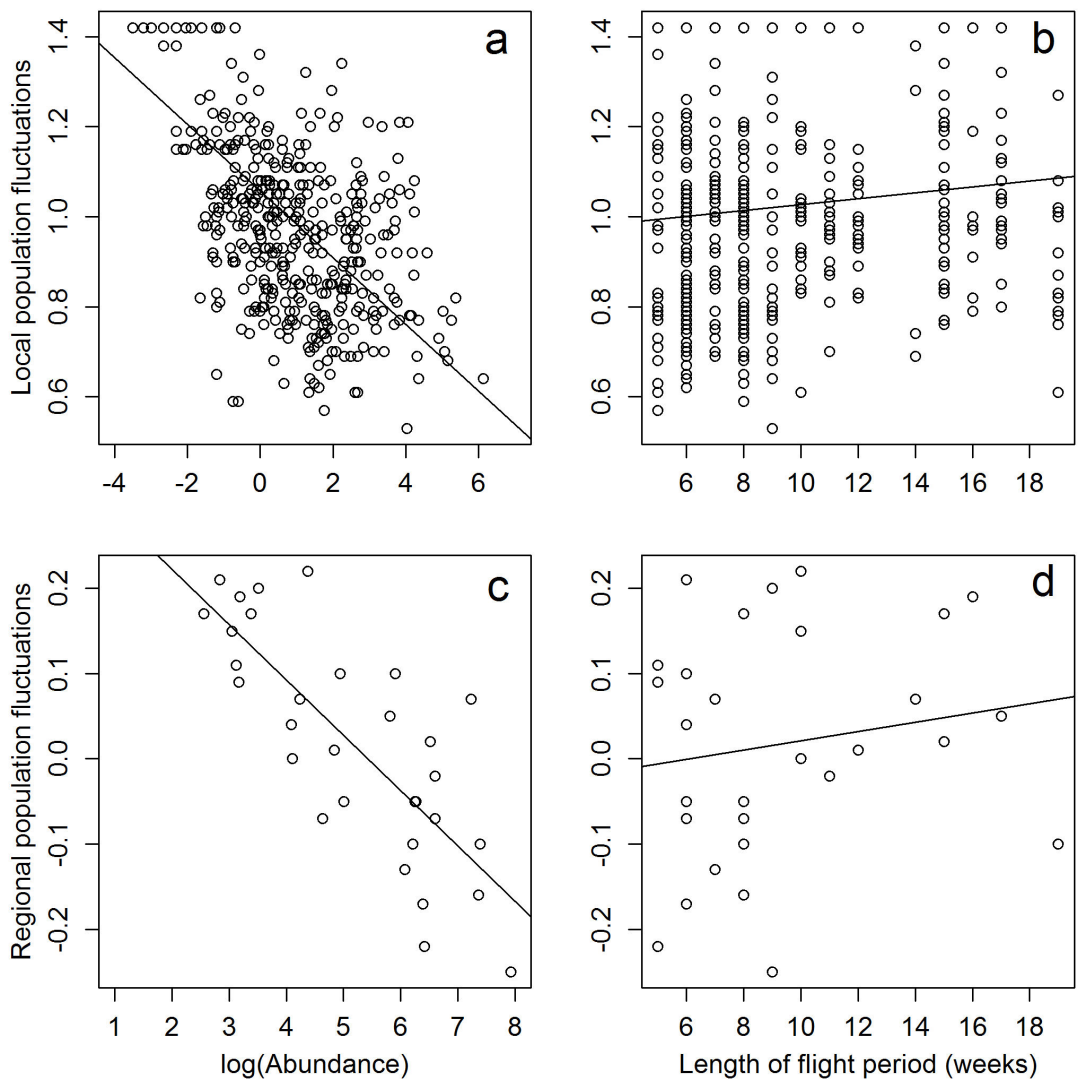
Local and regional butterfly population fluctuation in relation to abundance (a and c, respectively) and length of flight period (b and d, respectively).

At the local and regional level, population fluctuations increased with increasing length of the flight period and decreased with increasing abundance (Table 1, Figure 2a-d). Population fluctuations were not related to mobility, diet specialisation and distance to northern edge.

**Table 1:** Parameter estimates and standard error (SE) for the final models of butterfly population fluctuations (local and regional) and synchrony in relation to species traits and abundance.

	Local population fluctuations		Regional population fluctuations		Synchrony	
	Estimate (SE)	ΔAIC	Estimate (SE)	ΔAIC	Estimate (SE)	ΔAIC
log(Abundance)	-0.14 (0.0074)	260.4	-0.10 (0.015)	29.4	0.13 (0.036)	10.2
Length of flight period	0.04 (0.0071)	15.9	0.024 (0.015)	0.90	-	
Diet specialisation	-		-		-0.18 (0.09)	2.00

All continuous explanatory variables were standardised. ΔAIC = change in AIC when removing the variable from the final model.

**Figure 3:**
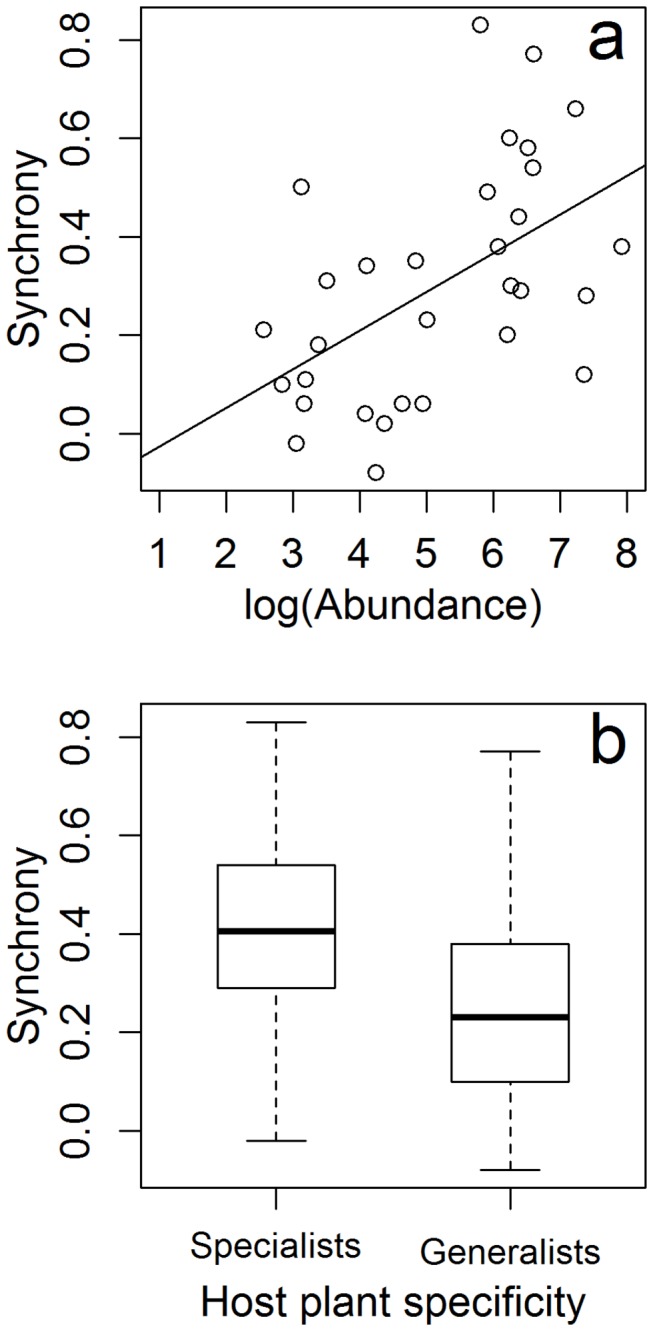
Synchrony of butterfly populations in relation to a) abundance and b) diet specialisation. The box plot indicates the smallest observation, lower quartile, median, upper quartile, and largest observation.

Among the study species, there was generally a tendency for fluctuating synchronously; the synchrony was positive for 29 out of the 31 species, and the mean value was 0.30. The degree of synchrony between local populations was higher for diet specialists than for diet generalists and increased with increasing abundance (Table 1, Figure 3). Synchrony in population fluctuations were not related to mobility or length of flight period. 

In an additional analysis excluding species with two generations per year, performed to ensure the robustness of our main approach, the results remained qualitatively similar (Table S2), i.e. the same species traits were important for population fluctuations and synchrony. 

## Discussion

This study provides a multiple-species test of associations between species-specific traits and population fluctuations. We demonstrate that (i) between-year population fluctuations have higher amplitude for species with longer flight periods and, (ii) diet specialists have fluctuations that are more synchronous among sites compared with diet generalists. However, for mobility and the distance to the northern distribution edge we did not find any of the expected relationships. 

We show that species with a long flight period fluctuated more than species with short flight periods, both at the local and regional level, in accordance with our hypothesis. However, in contrast, mobility did not explain population fluctuations. At the locality level, a longer flight period may increase the frequency of movements in and out of the sites. Fluctuations could be generated by individuals who track resources by dispersing in and out from sites, and by variation in reproduction rate and mortality. In the former case, the changes in the number of individuals occur at the local scale, while the number of individuals at the regional scale does not necessarily change. In the latter case, however, the population fluctuations occur at least to some extent also at the regional scale. Species with long flight periods may track the spatial fluctuations of nectar sources to a greater extent, and might aggregate to feed and reproduce far from the site where they hatched [26]. 

Presumably, resources for adults in terms of flower density are more variable than larval resources (host plants), and may result in larger population fluctuations in species with long flight periods. There was also a positive relationship between fluctuation width and length of flight period at the regional scale, even though weak as judged by the low ΔAIC (Table 1). This relationship may be due to that species with a long flight period to a higher extent migrate into the study area from adjacent areas increasing their fluctuations. Such events can occur in certain years with large populations and favourable weather conditions [27]. 

Species with a short flight period might be expected to have relatively low risks of local extinction, since the populations are less variable. However, this is opposite to the result in a long-term study, where butterflies with a short flight period had the highest local extinction rate [28]. If mobility is the cause of the wider fluctuations in our study this could also result in a stronger rescue effect which decreases the extinction risk, overriding the expected effect of wider fluctuations.

We show that species specialised to one or a few host plant genera have fluctuations that are more synchronous among sites than for those using many plant species, in accordance with our hypothesis. Since metapopulations with synchronous local population fluctuations are more extinction-prone [29], this may explain why diet specialists go regionally extinct more often compared to diet generalists [30]. Three mechanisms have been proposed to explain population synchronisation: dispersal, correlated environmental factors, and correlated trophic interactions [4]. The pattern in synchrony we found is likely related to the correlation in environmental factors, i.e. in factors affecting population growth (e.g. weather conditions, predation, parasitism, and diseases [31]). Indeed, population fluctuations can be more synchronous for species that are restricted to the same resources at every site, rather than for those utilising several resources, and possibly different resources at different sites [32]. In butterflies, larval mortality is often severe, and has therefore great impact on population growth [33]. It may vary more independently over time among local populations living on different host plant species than among those living on a single host plant species.

Species with a high abundance had narrower population fluctuations and more synchronised population fluctuations than species with low abundance (Figure 2). Species abundance strongly affects the level of population fluctuations and synchrony because stochasticity has greater influence when abundance is low [3]. We found no relation to the distance to the northern limit of their range, neither on population fluctuations nor on synchrony. However, such a relationship has been found previously and may be a result of that species close to their distribution limit are more sensitive to fluctuations in temperature and precipitation [11,12]. In the study area, all species close (

< 100 km) to their northern edge were very rare (<3 sites) and were not included in the statistical analysis, which might explain why we did not find this relationship. 

Our abundance estimate does not necessarily only reflect the true abundance, but can be influenced by weather conditions and visitation frequency. Especially for species with short flight periods, it may be that the peaks of their abundance are missed in our sampling. This would tend to increase the observed variability for species with short flight periods. However, this was the opposite to what was actually observed in this study, so this potential bias did not seem to have affected the outcome. Also varying weather conditions at the sampling event may tend to increase population fluctuation estimates but this gives rise to increased errors in general, but there is no reason to believe that they would give rise to a bias in any certain direction.

### Implications for conservation

We found that local populations of diet specialists are more synchronised than populations of generalists. This may explain why many previously widespread and common diet specialists have now disappeared from parts of their distribution area in Europe [34]; if synchrony is larger in diet specialists, these species are at higher risk of disappearing from larger areas at one time than species that occur on a larger number of host plants. If the fluctuations are synchronous over large areas, it is less likely that decreasing local populations will recover due to immigration. If population fluctuations will increase in the future, as a result of an increasing frequency of weather extremes [35], diet specialists may continue to decrease. Generalists may to some extent be better buffered against environmental extremes as these species can utilise a wider range of resources (more host plants). Therefore, if everything else is equal, diet specialists are more often threatened and require more conservation efforts than generalists.

## Supporting Information

Table S1 **The 31 studied butterfly species and the analyzed data.**
(DOCX)

Table S2 **Results from the linear models.**
(DOCX)
