# Supplementary material for: Population Fluctuations and Synchrony of Grassland Butterflies in Relation to Species Traits
Source: PLoS One. 2013 Oct 22;8(10):e78233. doi: 10.1371/journal.pone.0078233 (PMC3808534; doi:10.1371/journal.pone.0078233)
Supplement: Table S1 — The 31 studied butterfly species and the analyzed data. (DOCX) [file pone.0078233.s001.docx]

| Table S1. The 31 studied butterfly species and the analyzed data. Species are sorted in alphabetic order. See methods for further details. | | | | | | | | |
| --- | --- | --- | --- | --- | --- | --- | --- | --- |
| Species | Family | Abundance^a^ | Mean CV^b^ | Synchrony | Host plant specificity | Mobility | Length of flight period (weeks) | Distance to edge of northern range (km) |
| *Aglais urticae* | Nymphalidae | 335 | 0.05 | 0.83 | 1 | 3 | 17 | 1250 |
| *Anthocharis cardamines* | Pieridae | 103 | -0.07 | 0.06 | 1 | 2 | 8 | 1250 |
| *Aphantopus hyperanthus* | Nymphalidae | 2759 | -0.25 | 0.38 | 2 | 1 | 9 | 588 |
| *Aporia cratægi* | Pieridae | 17 | 0.21 | 0.1 | 2 | 3 | 6 | 1000 |
| *Argynnis adippe* | Nymphalidae | 60 | 0.04 | 0.04 | 1 | 2 | 6 | 888 |
| *Argynnis aglaja* | Nymphalidae | 594 | -0.17 | 0.44 | 1 | 2 | 6 | 1250 |
| *Argynnis paphia* | Nymphalidae | 739 | -0.07 | 0.77 | 1 | 2 | 6 | 826 |
| *Boloria selene* | Nymphalidae | 1380 | 0.07 | 0.66 | 1 | 1 | 7 | 1250 |
| *Brenthis ino* | Nymphalidae | 515 | -0.05 | 0.60 | 2 | 1 | 8 | 1250 |
| *Callophrys rubi* | Lycaenidae | 497 | -0.10 | 0.20 | 2 | 2 | 8 | 888 |
| *Celastrina argiolus* | Lycaenidae | 61 | 0.00 | 0.34 | 2 | 3 | 10 | 1103 |
| *Coenonympha pamphilus* | Nymphalidae | 69 | 0.07 | -0.08 | 2 | 1 | 14 | 1250 |
| *Favonius quercus* | Lycaenidae | 33 | 0.20 | 0.31 | 1 | 3 | 9 | 389 |
| *Gonepteryx rhamni* | Pieridae | 1615 | -0.10 | 0.28 | 1 | 3 | 19 | 888 |
| *Inachis io* | Nymphalidae | 733 | -0.02 | 0.54 | 1 | 3 | 11 | 1103 |
| *Leptidea sinapis* | Pieridae | 21 | 0.15 | -0.02 | 1 | 2 | 10 | 1250 |
| *Lycaena phlaeas* | Lycaenidae | 126 | 0.01 | 0.35 | 1 | 2 | 12 | 1250 |
| *Lycaena virgauraæ* | Lycaenidae | 613 | -0.22 | 0.29 | 1 | 2 | 5 | 888 |
| *Maniola jurtina* | Nymphalidae | 1574 | -0.16 | 0.12 | 2 | 2 | 8 | 695 |
| *Melitæa athalia* | Nymphalidae | 369 | 0.10 | 0.49 | 2 | 1 | 6 | 1250 |
| *Nymphalis antiopa* | Nymphalidae | 24 | 0.19 | 0.11 | 2 | 3 | 16 | 1250 |
| *Ochlodes sylvanus* | Hesperidae | 435 | -0.13 | 0.38 | 2 | 2 | 7 | 1093 |
| *Pieris napi* | Pieridae | 677 | 0.02 | 0.58 | 1 | 3 | 15 | 1250 |
| *Plebejus argus/idas* | Lycaenidae | 140 | 0.10 | 0.06 | 2 | 1 | 6 | 1103 |
| *Polygonia c-album* | Nymphalidae | 29 | 0.17 | 0.18 | 2 | 3 | 15 | 888 |
| *Polyommatus amandus* | Lycaenidae | 150 | -0.05 | 0.23 | 2 | 2 | 8 | 1103 |
| *Polyommatus icarus* | Lycaenidae | 79 | 0.22 | 0.02 | 1 | 2 | 10 | 1250 |
| *Polyommatus semiargus* | Lycaenidae | 24 | 0.09 | 0.06 | 1 | 2 | 5 | 1250 |
| *Pyrgus malvae* | Hesperidae | 13 | 0.17 | 0.21 | 1 | 1 | 8 | 695 |
| *Satyrium w-album* | Lycaenidae | 23 | 0.11 | 0.50 | 1 | 3 | 5 | 493 |
| *Thymelicus lineola* | Hesperidae | 528 | -0.05 | 0.30 | 2 | 2 | 6 | 493 |

a The number of individuals observed divided by the number of survey events during the flight period of the species.

b Mean of CV calculated separately for each occupied site
