# Supplementary material for: Population Fluctuations and Synchrony of Grassland Butterflies in Relation to Species Traits
Source: PLoS One. 2013 Oct 22;8(10):e78233. doi: 10.1371/journal.pone.0078233 (PMC3808534; doi:10.1371/journal.pone.0078233)
Supplement: Table S2 — Results from the linear models. (DOCX) [file pone.0078233.s002.docx]

Table S2. Parameter estimates and standard error (SE) for the final models of butterfly population fluctuations (local and regional) and synchrony in relation to species traits and abundance. All continuous explanatory variables were standardised. ΔAIC = change in AIC when removing the variable from the final model. Butterfly species with two generations per year was excluded from the analyses.

|  | Local population fluctuations | | Regional population fluctuations | | Synchrony | |
| --- | --- | --- | --- | --- | --- | --- |
|  | Estimate (SE) | ΔAIC | Estimate (SE) | ΔAIC | Estimate (SE) | ΔAIC |
| log(Abundance) | -0.15 (0.0080) | 244.4 | -0.12 (0.014) | 33.7 | 0.10 (0.038) | 4.60 |
| Length of flight period | 0.04 (0.0078) | 18.1 | 0.029 (0.014) | 2.40 | - |  |
| Diet specialisation | - |  | - |  | -0.24 (0.09) | 4.70 |
